# Pharmacological Application of Antiradical Compound Properties

**DOI:** 10.1155/2013/989604

**Published:** 2013-11-07

**Authors:** Alethéa Gatto Barschak, Francieli Moro Stefanello, Claiton Leonetti Lencina, Filippo De Simone, Wilson João Cunico Filho

**Affiliations:** ^1^Universidade Federal de Ciências da Saúde de Porto Alegre, Rua Sarmento Leite 245, 90050-170 Porto Alegre, RS, Brazil; ^2^Universidade Federal de Pelotas, Centro de Ciências, Químicas, Farmacêuticas e de Alimentos, 96060-000 Pelotas, RS, Brazil; ^3^Department of Chemistry, Chemistry Research Laboratory, University of Oxford, Oxford OX1 3TA, UK

The reactive species are part of normal human metabolism and are involved in many physiological mechanisms. However, when reactive species are overproduced and antioxidant defenses are insufficient to control oxidation, an unbalance can occur, leading to cellular oxidative damage. This injury has been associated with many diseases including inflammation, cancer, cardiovascular disease, diabetes, and neurodegenerative diseases. 

The population is ageing and chronic diseases affect millions of people around the world. Thus more effective treatments are necessary to provide improved quality of life for individuals. In this context, there has been growing interest in research involving natural and synthetic antioxidants which can emerge as alternative therapy and/or prevention of various chronic degenerative diseases.

In this special issue, we presented 11 original research papers and 3 review articles that describe the antiradical compound properties. The original papers explored in this special edition include a wide variety of topics such as natural products against injury (A. A. Soares et al. and S. Murthy et al.), antioxidant properties of natural products (E. Gregoris et al., A. Hashim et al., and A. Zajdel et al.), effect of glutathione on immune system (D. Morris et al.), antioxidant mechanism of buckminsterfullerene C^60^ (V. A. Chistyakov et al.), and spin labeled analogues of anticancer drugs in prevention of injury (V. Gadjeva et al.). 

The review articles discuss the role of antioxidants in chronic diseases: cardioprotective effect of vitamins (R. Rodrigo et al.), antidiabetic potential of curcumin analogues (Y. Son et al.), and detrimental and protective effects of fructose (H. M. Semchyshyn).

We believe that a better understanding of action of natural and synthetic antioxidants may contribute to the development of a new therapeutic approach in many different diseases, improving the patient's quality of life.

